# Predicting the role of assistive technologies in the lives of people with dementia using objective care recipient factors

**DOI:** 10.1186/s12877-016-0314-2

**Published:** 2016-07-20

**Authors:** Stephen Czarnuch, Rose Ricciardelli, Alex Mihailidis

**Affiliations:** Department of Electrical and Computer Engineering and Discipline of Emergency Medicine, Memorial University, S.J. Carew Building, St. John’s, NL A1B 3X5 Canada; Department of Sociology, Memorial University, Arts and Administration Building, St. John’s, NL A1C 5S7 Canada; Department of Occupational Science and Occupational Therapy & Institute of Biomaterials and Biomedical Engineering, University of Toronto, 160-500 University Ave., Toronto, ON M5G 1V7 Canada; Toronto Rehabilitation Institute-University Health Network, 550 University Avenue, Toronto, ON M5G 2A2 Canada

**Keywords:** Assistive technology, Predictive model, Needs assessment, Technology acceptance, Canadian population

## Abstract

**Background:**

The population of people with dementia is not homogeneous. People with dementia exhibit a wide range of needs, each characterized by diverse factors including age, sex, ethnicity, and place of residence. These needs and characterizing factors may influence the applicability, and ultimately the acceptance, of assistive technologies developed to support the independence of people with dementia. Accordingly, predicting the needs of users before developing the technologies may increase the applicability and acceptance of assistive technologies. Current methods of prediction rely on the difficult collection of subjective, potentially invasive information. We propose a method of prediction that uses objective, unobtrusive, easy to collect information to help inform the development of assistive technologies.

**Methods:**

We develop a set of models that can predict the level of independence of people with dementia during 20 activities of daily living using simple, objective information. Using data collected from a Canadian survey conducted with caregivers of people with dementia, we create an ordered logistic regression model for each of the twenty daily tasks in the Bristol ADL scale.

**Results:**

Data collected from 430 Canadian caregivers of people with dementia were analyzed to reveal: most care recipients were mothers or husbands, married, living in private housing with their caregivers, English-speaking, Canadian born, clinically diagnosed with dementia 1 to 6 years prior to the study, and were dependent on their caregiver. Next, we developed models that use 13 factors to predict a person with dementia’s ability to complete the 20 Bristol activities of daily living independently. The 13 factors include caregiver relation, age, marital status, place of residence, language, housing type, proximity to caregiver, service use, informal primary caregiver, diagnosis of Alzheimer’s disease or dementia, time since diagnosis, and level of dependence on caregiver. The resulting models predicted the aggregate level of independence correctly for 88 of 100 total responses categories, marginally for nine, and incorrectly for three.

**Conclusions:**

Objective, easy to collect information can predict caregiver-reported level of task independence for a person with dementia. Knowledge of task independence can then inform the development of assistive technologies for people with dementia, improving their applicability and acceptance.

## Background

Dementia is a progressive, neurodegenerative clinical syndrome characterized by the deterioration of cognitive functioning [1]. Although the classic feature of dementia is memory loss, behavioural and psychological symptoms are also salient [2] and negatively impact the ability of people with dementia (PwD) to negotiate their environment and independently complete activities of daily living (ADLs) [3]. The lost independence further burdens family caregivers [4–6] who act as assistants for cognition prompting, remind and support PwD in the performance of ADLs [7], and experience increasing burden as the disease progresses [8]. In response, assistive technologies (ATs), referring to devices designed to enable people with disability to function more independently [9], have been developed to support PwD and their caregivers. Such ATs, Pollack [10] argues, help people in three ways: “(1) by providing assurance that the elder is safe and is performing necessary daily activities, and, if not, alerting a caregiver; (2) by helping the elder compensate for [their] impairment, assisting in the performance of daily activities; and (3) by assessing the elder’s cognitive status” (p. 12). If effective, AT may help reduce the cost of care [9], decrease caregiver burden [11–13], promote independence and autonomy, and increase quality life for PwD [11, 13–15]. Additionally, AT may promote aging in place; thereby delaying the transition of PwD into formal care facilities and instead help them maintain some level of independence while living in the community [16]. Generally PwD prefer to age in place because it allows for their autonomy, independence, and connection to loved ones to be maintained [17, 18]. Yet little is known about how AT can be used to facilitate this practice [19].

In response, a user-centered design philosophy [20] has emerged among AT developers (e.g., [15, 21, 22]) to better understand the needs of PwD, incorporate these needs into AT design, and ultimately increase device acceptance and adoption among users [23]. User needs form the foundation of this design philosophy, including the characteristics of the users and their environments [13, 20, 23], which motivates more research on understanding user needs and how AT can best support PwD [24]. Researchers have found that internal personal features (e.g., expectations and self-esteem) [25] along with personal capabilities (e.g., cognitive abilities or deficits, attitudes toward technology) [9] are linked to AT adoption. Others document the role of environmental factors, (e.g., social setting, available infrastructure) [9, 25] to AT adoption and use. Limited understandings of the costs and availability of AT [19], in conjunction with the belief that technology cannot assist or is not appropriate for particular tasks [26] are also identified barriers to AT use.

Knowledge of factors affecting AT adoption will improve the likelihood of AT acceptance when considered during the design stage. However, to our knowledge, researchers employing a user-centred design philosophy have not considered the comprehensive needs of the target population. Rather, they tend to use a subset of user needs—just as we did in our previous work [13]. Developers of AT intended to support PwD often develop technologies without predicting the likelihood of the AT being adopted. To respond to these lacunae in the literature, we administered a national survey to family caregivers of PwD across Canada to characterize the comprehensive needs of PwD and their caregivers. We then analyzed the data collected via the survey to develop a predictive model of AT appropriateness with respect to daily task support.

### Factors characterizing people with dementia

Researchers reveal that demographic factors like age and gender can stratify the expected prevalence of dementia, demonstrating the heterogeneity of the population [27–30]. Alzheimer’s disease and other dementias, for example, are more common among women (e.g., in the United States nearly two of every three PwD are women [27]). Some argue this is because women live longer than men [28, 29]. In a more recent global analysis, researchers found that the prevalence of dementia doubled with every 5.5 to 6.7 years of age depending on the region [30]. They also noted that sex has an independent effect on the prevalence of dementia in all areas except North America and Asia Pacific [30]—supporting that the population of PwD is not homogeneous.

The population of PwD is also stratified by place of residence. For example, persons diagnosed with dementia living in the United States reported living in a nursing home or care facility (44 %) [27], in the community with another person (42 %), or alone in the community (15 %) [27, 31, 32]. These findings are similar for Canadian community-dwelling PwD where approximately 20 % to 30 % live alone [33]. Outside North America the place of residence of PwD varies considerably, with an estimated one-third to one-half of PwD living in residential care [30].

The impact of place of residence on PwD can be substantial. Researchers suggest PwD living alone in the community are more likely older, poorer, female, and more cognitively capable than those living with others [31]. They also appear more prone to harm requiring emergency attention [34] and malnourishment [35], but have less difficulty completing ADL [32]. Qualitative researchers find PwD living alone value their independence [36] and will adapt as necessary to remain at home as long as possible [36, 37]. Webber et al. [31] considered the impact of living alone on support service use, finding PwD living alone are more likely than those who live with others to use either in-home services (e.g., housekeeping, meals on wheels), or no services at all. Gaugler et al. [38] further noted that the use of in-home services by PwD results in a delay in institutionalization. Biegel et al. [39], in a study in the United States, classified support services for community-dwelling PwD based on the location of service provision: in-home (e.g., housekeeping); out-of-home (e.g., transportation); both in- and out-of-home; and none. They found PwD using in-home or no services had higher functional impairment, inadequate informal supports, and caregivers who reported a higher level of emotional strain.

In summary, we highlight that many factors stratify the heterogeneous population of PwD. Specifically, we note from the literature that age, sex, ethnicity, place of residence, socioeconomic status, and the use of support services have been identified as relevant stratifying factors. Accordingly, we seek to determine the utility of these and potentially other factors for characterizing the AT needs of PwD. Such factors may facilitate the prediction of specific tasks that PwD and their caregivers struggle with and the likelihood of adopting an AT designed to support those tasks.

### Predicting needs and the likelihood of adoption

Davis [40, 41] considered predicting the likelihood of users accepting general technologies (and eventually computers – at a time when computers were considered technological innovations) using a scale with two perceived variables, usefulness and ease of use. Out of Davis’ seminal work [41] emerged the Technology Acceptance Model (TAM) which underwent several evolutions [42–44], specifically as a tool to predict general acceptance of technology. The predictive capability and explanatory potential of the TAM have since been criticized [45–48], and some have considered that research on improving the model may be saturated [49]. In particular, the model’s use of subjective data is a noted significant limitation [47, 50, 51]. Less than a decade later, Day and Jutai [52] developed the Psychosocial Impact of Assistive Devices Scale (PIADS) for assistive technologies, in contrast to the TAM which focused on general technologies. Similar to TAM, PIADS was criticized for relying on subjective information in order to be effective and, thus, ultimately having limited predictive capabilities [49]. In an attempt to address these challenges, Zhang et al. [25], developed and compared several models based on care recipient factors that could predict the likelihood of a phone-based video streaming AT being adopted for PwD. They determined that seven care recipient features affect the predictive model: sex; living arrangement; MMSE score; broadband connection availability; age; mobile device reception; and carer involvement. Although the predictive capabilities of the model were strong, they were limited to a single AT and thus their work is not broadly applicable. Extending this work, we strive to develop generalizable predictive models for AT using objective, unobtrusive information about PwD.

### Linking characteristics to needs to acceptance

To our knowledge, scholars have yet to develop a predictive model using objective characteristics of PwD to identify their general AT needs. In response, we quantify objective factors, drawn from the literature, that characterize the current Canadian population of PwD (Objective 1). We then develop a set of models to predict the level of difficulty a care recipient has with different ADL based on the characterizing factors (Objective 2). Next, we determine the role of AT in supporting the ADL needs of PwD. From these predictive models, we inform AT designers of the tasks that are most appropriate for AT interventions to increase AT adoption (Objective 3). We propose the use of objective care recipient factors (e.g., demographics) provided by caregivers for our models because these data are easy to identify, non-invasive, and unobtrusive to collect. As such, caregiver reports on their care recipient’s ability to complete ADL are used in the resulting predictive models alongside simple, objective care recipient characteristics. The end goal is to provide AT developers with knowledge of the ADL PwD require help with the most.

## Methods

This study is part of a larger project initiated to inform the development of generalizable AT design guidelines for PwD.

### Participants and recruitment

Participant recruitment occurred across Canada through: 1) National, provincial and regional not-for-profit organizations; 2) Community support groups; 3) Hospitals and treatment clinics; 4) medical practitioners’ clinics; and 5) radio. Recruitment methods across nine provinces included the distribution of paper and electronic recruitment flyers; links to the online questionnaire on organizational websites and electronic mailing lists; and snowball sampling/word-of-mouth. Inclusion criteria for the study mandated participants were Canadian and provided unpaid care for a person with AD or another type of dementia.

### Questionnaire design

A 159-item questionnaire, using a combination of 89 constructed items and 70 items from existing validated scales [53–58], was created in six thematic sections. The full questionnaire is available online in English and French [59, 60] and designed to be analyzed in and across subsections. To satisfy the objectives of the current study we used 13 demographic questions from Section C: *Care Recipient Information,* and the Bristol ADL Scale [54]. The questionnaire, including constructed questions, was first piloted on experts in the fields of dementia care and AT (*n* = 7). After incorporating any recommended changes, we piloted the questionnaire again on five regional Alzheimer’s Societies and local centres for support and education, always making modification as necessary. The final survey is translated into French (Canadian).

### Procedure

University of Toronto (REB#12-044) and Memorial University (ICEHR# 20140464-EX) granted ethics clearance for the study. The questionnaire was posted online in English [59] in March, 2013 and French (Canadian) [60] in September, 2013. The research team also distributed hard copies of the questionnaire and a toll-free telephone number to participating organizations in order to accommodate participant preferences. Prior to completing the questionnaire, a summary of the study was given to participants who then provided informed consent. After completing the questionnaire, participants could choose to provide their contact information if they were interested in continuing their involvement through future stages of the study.

### Model development

**Fig. 1:**
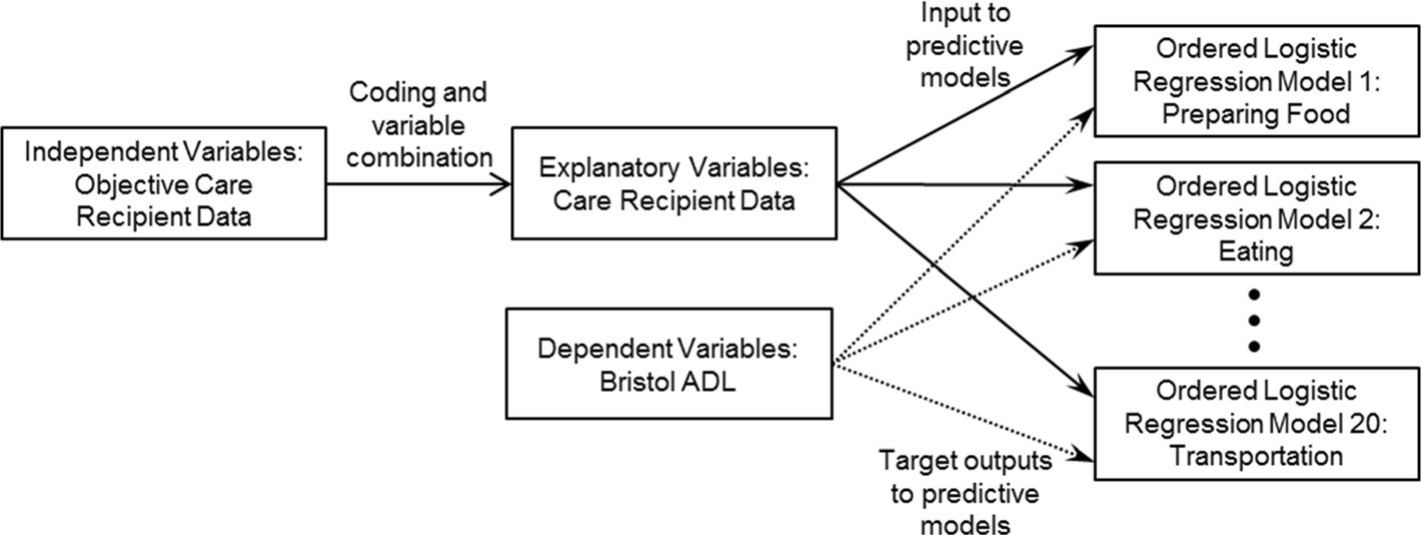
Workflow for the development of the set of twenty predictive models. The set of independent variables, coded as explanatory variables, along with the set of dependent variables, are used to develop the set of twenty predictive models

According to the workflow shown in Fig. 1, we developed a set of predictive models. We outline each step of this process in detail in Objective care recipient demographic data and Predictive modelling.

#### Objective care recipient demographic data

We draw from the demographic data in Section C: *Care Recipient Information* to characterize the current Canadian population of PwD (Objective 1). Within the demographic responses, categories were grouped if they were thematically related and correlated in the statistical modeling (e.g., same household and same building) or if they were not thematically linked but represented less than 5 % of the total responses.

#### Predictive modelling

##### Model variables

To develop the set of models that predict the level of difficulty care recipients will have with different ADL (Objective 2), we first define the twenty ADL (Bristol ADL [54]) as dependent variables (Table 1). Responses to these variables represent caregiver opinions of the care recipients’ abilities to complete these ADL. We then set the independent variables (Table 2) as the participant responses to the 13 objective care recipient demographic questions from Section C.

**Table 1:** Dependent variables

Number	Dependent variable	Number	Dependent variable	Number	Dependent variable	Number	Dependent variable
1.	Preparing Food	6.	Hygiene	11.	Mobility	16.	Housework/Gardening
2.	Eating	7.	Teeth Cleaning	12.	Orientation – Time	17.	Shopping
3.	Preparing a Drink	8.	Bathing/Showering	13.	Orientation – Space	18.	Finances
4.	Drinking	9.	Toileting	14.	Communicating	19.	Games/Hobbies
5.	Dressing	10.	Transferring	15.	Telephone	20.	Transportation

**Table 2:** Independent variables

Care recipient (CR) demographic information	Short name	Data type
What is your relationship to the CR?	Relation	Nominal
How old in years is he or she?	Age	Continuous
What is the CR’s marital status?	Marital	Nominal
Was the CR born in Canada or outside Canada?	Birthplace	Nominal
What is the CR’s primary language?	Language	Nominal
In what type of housing does the CR live?	Housing	Nominal
How close to you does the CR live?	Proximity	Ordinal
During the past 12 months, how frequently did the CR receive help from paid professionals or organizations	Paid care	Ordinal
Would you say that, other than professional care, the CR considers you to be his or her primary caregiver?	Primary	Nominal
Has the CR been clinically diagnosed with AD?	AD	Nominal
If no, has the CR been clinically diagnosed with another form of dementia?	Dementia	Nominal
If the CR has been clinically diagnosed with AD or another form of dementia, approximately how long ago was this diagnosis made?	Duration	Ordinal
On a scale of 0 to 10, 0 meaning “not dependent at all” and 10 meaning “completely dependent,” how dependent would you say the CR is on you for help?	Independence	Interval

##### Explanatory variables

Of the 13 independent variables 12 are discrete (i.e., ordinal or nominal) which makes their literal value difficult to compare within response categories. Accordingly, each independent variable, with *l* response levels, was coded into an (*l*-1)-tuple dummy variable. A variable with four response levels, for example, was represented by (0,0,0) for the baseline response level and the 3-tuple (1,0,0), (0,1,0), (0,0,1) for the three comparison response levels. In some cases, when multiple levels had similar effects on the outcome, response levels were combined. For example, the proximity baseline variable was composed of response level one (same house) and response level two (same building). Using this coding scheme, we developed the explanatory variables (Table 3) and selected the baseline group as the first response category group.

**Table 3:** List of baseline and comparison explanatory variables, and the scale items included in each

Scale item short name		Baseline variable label	Baseline variable response levels	Comparison variable label	Comparison variable response levels
1.	Relation	Male	1,3,5,7	Female	2,4,6,8
				Other	9,10,11
2.	Age	less than 70	-	70–79	-
				80–89	-
				90+	-
3.	Marital	Married, divorced, widowed	1,3,5	Common-law, single	2,6
				Separated	4
4.	Birthplace	In Canada	1	Outside Canada	2
5.	Language	English	-	French	-
				Other	-
6.	Housing	Private, institution, other	1,3,4	Supportive	2
7.	Proximity	Same house/same building	1,2	Less than 1 h away	3,4,5
				1 h or more away	6,7
8.	Paid care	Daily	1	2–3 times per week	2,3
				Less than weekly	4,5,6,7
9.	Primary	Yes/Unknown	1,3	No	2
10.	AD	Yes	1	No	2
11.	Dementia	Yes	1	No	2
12.	Duration	Less than 1 year	1	1–6 years	2,3
				6+ years	4,5
				N/A^a^	6
13.	Independence	Not dependent at all	1	Slightly dependent	2,3,4,5
				Dependent	6,7,8,9
				Very dependent	10,11

^a^Note: N/A includes “Not Sure” responses from questions 10 and 11 (clinical diagnoses of AD or dementia)

##### Modelling

The 20 dependent variable responses belonged to the ordered set, $R\in \left\{a,b,c,d,e\right\}$, where *a* to *d* represented an ordered range from “independent” to “completely dependent”, and *e* represented “Not Applicable”. We defined $k\in \left\{1,2,3,4,5\right\}$, where $\left(1,2,3,4,5\right)=\left(a,b,c,d,e\right)$, assigning a numerical value to each response category. An ordered logistic regression model [61], selected for its suitability to ordinal variables, was created for each of the 20 dependent variables using the coded explanatory variables. Each model estimates the proportion of responses within each category (a) through (e) for each dependent variable:

1 $$ logit\left( Pr\left({Y}_i\le k\right)\right)={n}_k-\beta {X}_i $$

where ${Y}_i$ is the response of the i^th^ respondent; $Pr\left({Y}_i\le k\right)$ is the probability that the response is in the *k*^*th*^ or lower category; ${n}_k<{n}_{k+1}$ are the response category boundaries; *β* is the vector of twenty-six regression coefficients (unique per ADL, corresponding to the comparison variables); and ${X}_i$ is the vector of explanatory variables. The regression model for any $c\in \left\{1,2,3,4,5\right\}$ is then:

2 $$ Pr\left({Y}_i\le c\right)=\frac{e^{\left({n}_c-\beta {X}_i\right)}}{1+{\displaystyle {\sum}_k}{e}^{\left({n}_k-\beta {X}_i\right)}} $$

## Results

### Participants

To characterize the current Canadian population of PwD (Objective 1), we look at the demographic information of 430 Canadian caregivers who provide unpaid care to PwD and voluntarily completed the survey. Of these, 311 (72.3 %) respondents completed it online and 119 (27.7 %) on paper (five in French). Of the participants 79.1 % self-reported as female (*n* = 340), 19.8 % male (*n* = 85) and 1.2 % (*n* = 5) did not report their gender. Their ages ranged from 20 to 94 years (*n* = 428, M = 62.75, SD = 12.67, missing data = 2). The care recipient group were reported to be 40.2 % females (*n* = 173), 40.4 % males (*n* = 174), 8.1 % (*n* = 35) of unknown sex (e.g., sibling), and 11.1 % (*n* = 48) unreported. Care recipient ages ranged from 45 to 98 (*n* = 389, M = 78.52, SD = 10.21). Table 4 presents the dominant care recipient demographic trends for each questionnaire item.

**Table 4:** Care recipient demographics as reported by their care giver

Category	Item	Number of respondents	Percentage
Relation to Caregiver	Father/Father-in-law	31	7.2
	Mother/Mother-in-law	112	26.0
	Husband	143	33.3
	Wife	61	14.2
	Other	35	8.1
	Missing	48	11.2
Marital Status	Married	229	53.3
	Widowed	117	27.2
	Other	43	10.0
	Missing	41	9.5
Birthplace	In Canada	265	61.6
	Not in Canada	126	29.3
	Missing	39	9.1
Primary Language	English	339	78.8
	French	20	4.7
	Other	29	6.7
	Missing	42	9.8
Type of Housing	In private household	315	73.3
	Institution al care facility / Supportive housing / Other	69	16.0
	Missing	40	9.3
Proximity to Caregiver	Same household / Same building	269	62.6
	Less than 1 h by car	110	25.6
	More than 1 h by car	14	3.3
	Missing	37	8.6
Paid Care	Daily	84	19.5
	2–3 times per week	103	24.0
	Less than weekly	92	21.4
	Never	107	24.9
	Missing	44	10.2
Informal Primary Caregiver	Yes	341	79.3
	No/Unsure	50	11.6
	Missing	39	9.1
Clinical diagnosis	Alzheimer’s disease	274	63.7
	Other dementia	91	21.2
	No/Unsure	9	2.1
	Missing	56	13.0
Time since diagnosis	Less than 1 year	41	9.5
	1–6 years	256	59.5
	6+ years	76	17.7
	Unknown	8	1.9
	Missing	49	11.4
Independence	Not dependent at all (0–2)	21	4.9
	Slightly dependent (3–5)	62	14.4
	Dependent (6–8)	157	36.5
	Very dependent (9–10)	148	34.4
	Missing	42	9.8

### Predictive models

**Fig. 2:**
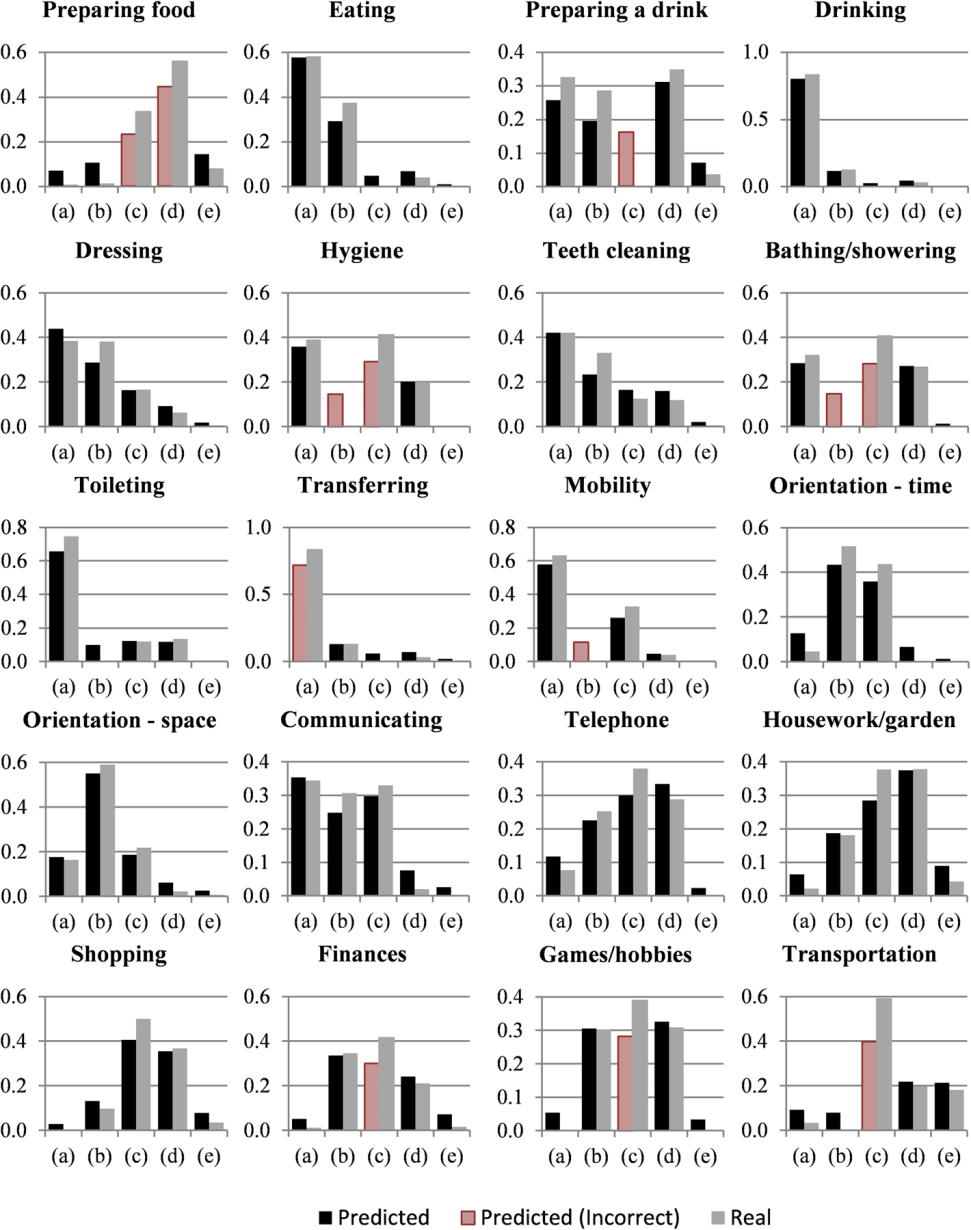
Comparison of the proportions of predicted and real responses. Responses categories range from Independent (a) to Completely Dependent (b), and Not Applicable (e). Predicted responses that are not within 10 % of the proportion of actual respondents are highlighted as incorrect predictions

To predict the level of difficulty care recipients have with ADL (Objective 2), we created a multinomial logistic regression model for each of the 20 ADL (see Table 1 for ADL). Each of the 20 models predicts one of five responses ((a) through (e)) resulting in 100 total response categories. We present statistics on the relative quality of the models, including Akaike Information Criterion (AIC), in Table 5. The mean of the difference between real and predicted responses, (a) through (e), for all twenty ADL are 0.0461, 0.0659, 0.0786, 0.0312, and 0.0233 with variances of 8.47 × 10^−4^, 2.17 × 10^−3^, 2.53 × 10^−3^, 7.13 × 10^−4^, and 3.28 × 10^-4,^ respectively. These mean differences represent the average differences between the proportion of real and predicted responses for each of the twenty ADL. This means, for example, that the models were able to predict response (e) most accurately (mean difference 0.0233), and (c) least accurately (mean difference 0.0786). As evidenced in Fig. 2, the models correctly predict the proportion of respondents in each category (a) through (e) across the 20 ADL to within 10 % of the total respondents in 88 of the 100 total response categories (solid black, Fig. 2). For example, in the telephone ADL all five predicted responses were within 10 % of the actual proportion of responses. In 12 of the 100 total response categories, the difference between the predicted and actual proportion of respondents was greater than 10 % of the total respondents (hash marked, Fig. 2). For example, in the preparing food ADL, predicted responses (c) and (d) provide incorrect predictions that are more than 10 % lower than the real responses.

**Table 5:** The residual deviance and AIC for the twenty multinomial logistic regression models

ADL	Preparing food	Eating	Preparing a drink	Drinking	Dressing	Hygiene	Teeth cleaning	Bathing/Showering	Toileting	Transferring
Residual Deviance	1121	852	1206	535	1039	1058	1080	1084	807	713
AIC	1183	914	1268	597	1101	1120	1142	1144	869	773
ADL	Mobility	Orientation – Time	Orientation – Space	Communicating	Telephone	Housework/Gardening	Shopping	Finances	Games/Hobbies	Transportation
Residual Deviance	818	966	906	1099	1113	1118	999	1102	1073	1147
AIC	878	1028	966	1161	1175	1180	1059	1164	1135	1209

## Discussion

In characterizing Canadian PwD (Objective 1), most care recipients were mothers/mother-in-laws and husbands, married, and living in private housing along with their caregivers—all consistent with current conceptions of PwD cared for by family caregivers [27, 30–33]. Furthermore the majority were English-speaking, Canadian born, and had received a clinical diagnosis of AD and/or dementia from 1 to 6 years prior. Most caregivers reported their care recipient was dependent or very dependent on the care they provided, and the frequency of their use of professional services (ranging from never to daily use) was equally distributed. Given most resources available to support PwD in Canada, and that our recruitment tools (e.g., regional Alzheimer Societies), are predominantly in English, our sample may not fully-represent the underlying multicultural Canadian care recipient population (i.e., the sample is predominantly English-speaking). Further, recruitment resources are likely only used by people who have or are caring for a person with a diagnosis—persons targeted by the resources. This also helps explain why the vast majority of respondents had received a diagnosis over a year prior to their participation—they had time to find resources yet were not at a point (i.e., after 6 years with the illness) when they would likely need to shift to more formal caregiving processes and persons. The broad frequency of use of professional services suggests a diverse set of needs despite our respondents ranking their care recipient as dependent to very dependent and predominantly living in private housing. Both high dependence (or high impairment) and community-dwelling have suggested a bimodal usage of services: either substantially using services or not at all [31, 38, 39], which warrants future research into these relationships.

Inspection of the demographic information emergent from the data helps provide an understanding of Canadian PwDs and identifies limitations with our sample. For example, our sample’s limited multicultural representation motivates greater representation of the diverse Canadian population in future studies. To this end, we are currently translating the questionnaire with the end goal of garnering insight into the needs of the Chinese-Canadian community. These additional multicultural demographics may further strengthen our proposed models and respond to the needs of more Canadian PwD. In seeking to develop a set of models to predict challenging ADL for PwD based on characterizing factors (Objective 2), we found multinomial logistic models work well (88 of 100 response categories correctly predicted). The models predict caregiver responses at the extremes of the response continuum and do well estimating the overall distribution of responses, although a small number of predicted response categories (12 of 100) were outside our defined success threshold of 10 % of total respondents. Of the 12 cases, nine occurred within the preparing food and drink, hygiene, bathing/showering, mobility, and transportation tasks. In each, the models predicted (b) or (c) while the respondents selected the opposite responses (c) or (b). These middle response categories may be more challenging to predicte since the care recipients are transitioning toward needing more help in these stages. Care recipients in earlier stages (e.g., b) may recognize their needs, however it may be harder to contextualize the extent of those needs given the illness has yet to progress fully and everyone is adjusting to the diagnosis/health status. However, as the disease progresses and the care recipient’s needs become pronounced, the definition of need is clearer. Given the subjectivity inherent in need characterization juxaposed with the emotional components of caregiving, we do expect some ambiguity in responses. Accordingly, it is not surprising that the models predict responses (b) and (c) least accurately, both in application and statistically (see Predictive models). By looking at the aggregate prediction of (b) and (c), it is evident that these models accurately predict care reicipent needs during the transitory period of disease progression.

Ultimately, our interest is to help developers produce needed and utile AT that can support PwD (Objective 3). We draw on our findings to identify the ADL most PwD indicate needing assistance with to satisfy this objective. We do this by examining the proportion of “dependent” (c) and “very dependent” (d) responses for each task (visualized in Fig. 2). For example, almost 80 % of caregivers surveyed about their care recipient’s ability to prepare food indicate a high level of dependence completing this task. Hygiene, bathing/showering, telephone, housework/gardening, shopping, finances, games/hobbies, and transportation are similar. Most of these ADL are complex, or instrumental ADL, and our models had difficulty correctly predicting responses for six of these nine ADL. Perhaps, more complicated and broader tasks evoke responses that are more ambiguous. Combining categories (c) and (d), however, accounts for the models’ predicative inaccuracies here. In other words, although our models erroneously predicted the individual response categories (c) and (d) for several ADL, the majority of participant responses for the ADL were predicted correctly using the aggregate of (c) and (d). Thus, we propose using these models to identify the ADL that are most in need of AT for support based on the needs expressed by PwD. In our sample, the ADL preparing food, hygiene, bathing/showering, telephone, housework/gardening, shopping, finances, games/hobbies, and transportation are tasks where care recipients exhibit a high level of dependence on their caregiver. In this context, these daily tasks could benefit most from AT that could both support the independence of the PwD and relieve the burden experienced by the caregiver. Outside of our Canadian sample, using easy to collect, objective care recipient information with our models, AT developers may be able to identify the ADL that PwD are most dependent on their caregiver to complete. The development of AT that satisfy the needs of PwD must be centred on these ADL; those that are most in need of intervention.

## Conclusion

Technology developers should develop AT with knowledge of the independence and capabilities of PwD completing various ADL. However, such capabilities vary and their direct evaluation is time-consuming, subjective, and potentially invasive. We show how the task-based independence of PwD is shaped by 13 factors and argue these factors are more objective, easier to identify, and less invasive than a more direct evaluation of task-based independence. These 13 factors are: caregiver relation, age, marital status, place of residence, language, housing type, proximity to caregiver, use of professional service, informal primary caregiver, diagnosis of Alzheimer’s disease, diagnosis of dementia, time since diagnosis, and level of dependence on caregiver. We use these factors to develop a set of models that can predict the likelihood that PwD will require assistance during 20 ADL without direct evaluation of their actual capabilities. In this way, AT developers can simply collect objective data from a PwD and identify the ADL that require the most support. The social and quality of life implications of such developments are overwhelmingly positive. Given the realities of independence loss, reduced quality of life and burden for both PwD and their caregivers, the ability to target AT development in areas that offer the most assistance, taking into account characteristics of PwD, can potentially offer positive gains in the abilities of persons with dementia to age in place.

## Abbreviations

ADL, activity of daily living; AT, assistive technology; MMSE, mini–mental status examination; PIADS, psychosocial impact of assistive devices scale; PwD, people with dementia; TAM, technology acceptance model.
